# Signal of Acceleration and Physical Mechanism of Water Cycle in Xinjiang, China

**DOI:** 10.1371/journal.pone.0167387

**Published:** 2016-12-01

**Authors:** Guo-Lin Feng, Yong-Ping Wu

**Affiliations:** 1 College of Physics Science and Technology, Yangzhou University, Yangzhou, Jiangsu 225002, China; 2 National Climate Center, China Meteorological Administration, Beijing 100081, China; 3 Ecological Complexity and Modeling Laboratory, University of California Riverside, Riverside, CA 92521-0124, United States of America; Hangzhou Normal University, CHINA

## Abstract

Global warming accelerates water cycle with features of regional difference. However, little is known about the physical mechanism behind the phenomenon. To reveal the links between water cycle and climatic environment, we analyzed the changes of water cycle elements and their relationships with climatic and environmental factors. We found that when global warming was significant during the period of 1986-2003, the precipitation in Tarim mountains as well as Xinjiang increased rapidly except for Tarim plains, which indicated that there existed a signal of acceleration for water cycle in Xinjiang. The speed of water cycle is mainly affected by altitude, latitude, longitude, slope direction, and the most fundamental element is temperature. Moreover, according to Clausius-Kela Bai Lung relation, we found that the climate change induced the increase of temperature and accelerated the local water cycle only for the wet places. Our results provide a possible physical mechanisms of water cycle and thus well link the climate change to water circulation.

## Introduction

Water moves through the land and air, and impacts the ecological and biological systems [1–6]. The circulation mechanism in the water cycle include evaporation, transportation, condensation, precipitation, runoff and groundwater [1], and each of these components has its unique role [7–12]. The water cycle begins with the evaporation of the water in rivers, lakes and oceans and thus water turns into water vapor. Then the water condenses into clouds when it encounters the cold air. When enough water vapor condenses into the clouds, it will turn into either liquid water or ice. Finally, it falls back to the ground through precipitation, which will eventually evaporate again.

In the whole world, especially in arid and semiarid region, characteristics of water cycle change a lot as a result of intensive variation of nature environments and development of human society in recent decades [4, 8]. Previous studies showed that the results of the impaction of global warming on water cycle have regional differences [13]. More intense and short rainfall events lead to floods [14, 15] and more dry days in year which extended drought periods and thus induced droughts all over the world [16]. More and greater floods and droughts are causing a lot of socio-economic problems [17].

In Xinjiang, a region located in northwest China, wet-dry trend is becoming increasingly evident which has been attracted a huge amount of interests [10, 11, 18–20]. Signals of warming and wetting emerged in many places all over the world, as well as Xinjiang mountain areas under the global warming [21], and reversed with the drying in northwest China. However, the mechanism of water cycle in Xinjiang, China is far from being well understood. Especially, one may ask: (1) what are changing in water cycle elements in Xinjiang? (2) What are the main factors for acceleration of water cycle? (3) How do climatic and environmental factors act on the water cycle elements? To address these questions, the changes in water cycle elements and the relationship between them and climatic environmental factors are analyzed.

The rest of this paper is organized as follows. We firstly present the materials and methods used in this paper. The first one is the studied data and area with the complex coverage of vegetation and glaciers. And the second one is the method for analyzing mutation time and linear trend of precipitation. Then we reveal the signal of acceleration of water cycle in Xinjiang, the relationship between water cycle and environment factors and the physical mechanism behind the acceleration of water cycle. In the final part, we draw the main conclusions and discuss the potential investigations for future developments.

## Materials and Methods

### Studied area and data

**Fig 1:**
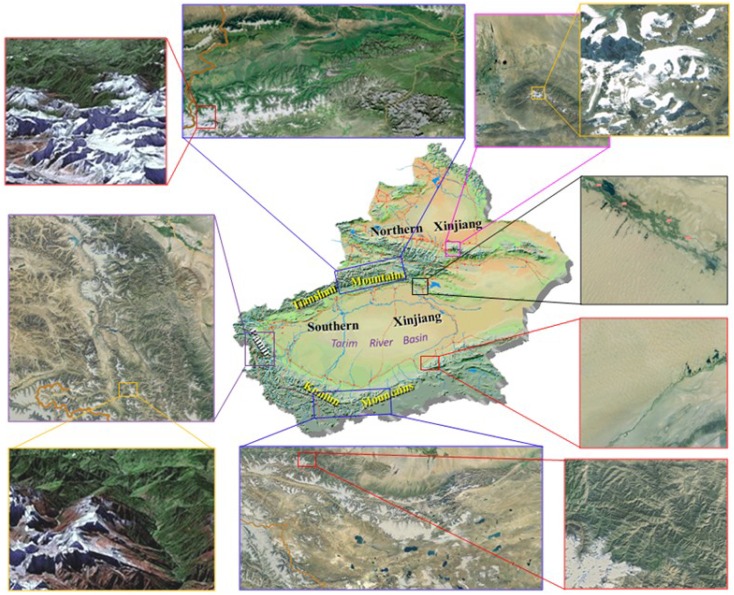
Geography and topography of Xinjiang, China. Mountains, such as Tianshan Mountains, Pamir, Kunlun Mountains, are covered by dense vegetation and glaciers. However, sparse vegetation and large tracts of desert present over plains, especially in Tarim River Basin. Map of Xinjiang is created by using ArcGIS which is an open access software (http://www.esrichina.com.cn/softwareproduct/ArcGIS/). Maps of small regions are drawn by Matlab R2015a. Lines are added by Microsoft Office Word 2007.

Xinjiang, which is divided into two parts by Tianshan mountains and the local climate system is extremely complex for the different underlying surface (Fig 1). There is abundant water over mountains resulted from the rich plants and glaciers, while scarce water over plains because of the little vegetation and a large area of Gobi desert. The temperature and precipitation data used in this paper are the observed monthly mean data from 60 Xinjiang meteorological and hydrological Stations and among them 26 stations from Tarim River Basin were chosen to determine the variation trend of precipitation over the mountains and plains respectively. Data can be accessed in the website: http://www.cma.gov.cn/.

### Analysis of mutation time of precipitation

In order to analyze the mutation time of the precipitation in Southern and Northern Xinjiang, cumulative valuation of modulus coefficient is used. It is defined as:

$$\begin{array}{c} \sum_{i=1}^{n}\left(\frac{x_{i}}{\bar{x}}-1\right), \end{array}$$ (1)

where *x*_*i*_ is the observation valuation and $\bar{x}$ is the mean valuation during a period. The change in the trend of the time series of cumulative valuation of modulus coefficient is usually used to judge the mutation time.

The water vapour net income is computed according to water vapour flux (water vapor flux is defined as the water vapor mass passing vertically through per unit square in per unit time) *Q*, which is expressed as follows [22, 23]:

$$\begin{array}{c} Q=\frac{1}{g}\left|\vec{v}\right|q\Delta l\cdot \Delta p, \end{array}$$ (2)

where *g* is the gravitational acceleration, $|\vec{v}|$ is the size of *u* wind or *v* wind, *q* is the specific humidity, Δ*l* is the length increment of the meridional or zonal side for some area, and Δ*p* is the height increment in pressure coordinates. When Δ*l* = 1*m*, the zonal and meridional components of *Q*, denoted by *Q*_*x*_ and *Q*_*y*_, respectively, are expressed as:

$$\begin{array}{c} Q_{x}=\frac{1}{g}\int_{p_{t}}^{p_{s}}qudp, \end{array}$$ (3)

and

$$\begin{array}{c} Q_{y}=\frac{1}{g}\int_{p_{t}}^{p_{s}}qvdp. \end{array}$$ (4)

The areas of zonal and meridional water vapour net input (WVNI) are expressed respectively as:

$$\begin{array}{c} \Delta Q_{x}=Q_{xW}-Q_{xE}=\frac{1}{g}\left[\int_{l_{W}}\int_{p_{t}}^{p_{s}}qudpdy-\int_{l_{E}}\int_{p_{t}}^{p_{s}}qudpdy\right]. \end{array}$$ (5)

and

$$\begin{array}{c} \Delta Q_{y}=Q_{yS}-Q_{yN}=\frac{1}{g}\left[\int_{l_{S}}\int_{p_{t}}^{p_{s}}qvdpdx-\int_{l_{N}}\int_{p_{t}}^{p_{s}}qvdpdx\right], \end{array}$$ (6)

where Δ*Q*_*x*_ and Δ*Q*_*y*_ represent the zonal and meridional WVNI, respectively; *Q*_*xW*_, *Q*_*xE*_, *Q*_*yS*_, and *Q*_*yW*_ represent the amount of water vapour transported through the West, East, South, and North boundaries of the area, respectively, within unit time; and *l*_*W*_, *l*_*E*_, *l*_*S*_, and *l*_*N*_ are the West, East, South and North boundaries of the area. As a result, horizontal WVNI is expressed as:

$$\begin{array}{c} \Delta Q=\Delta Q_{x}+\Delta Q_{y}. \end{array}$$ (7)

### R/S analysis

To catch the signal of acceleration of water cycle, the change trends of water vapor net income and other related components over Tarim River Basin such as precipitation and other related components in Southern and Northern Xinjiang and Tarim mountains and plains were analyzed, using R/S [24–26] and Linear Trend methods.

R/S analysis method is put forward by Hurst when he analyzed the Nile hydrological data [9]. The principle of the method is as follows. A time sequence is known to be

$$\begin{array}{c} X(t),\;t=1,2,\ldots , \end{array}$$ (8)

then its average value sequence is

$$\begin{array}{c} <X{>}_{\tau }=\frac{1}{\tau }\sum_{t=1}^{\tau }X\left(t\right),\;\tau =1,2,\ldots . \end{array}$$ (9)

The accumulative deviation is

$$\begin{array}{c} X\left(t,\tau \right)=\sum_{u=1}^{t}\left[X\left(u\right)-<X{>}_{\tau }\right], \end{array}$$ (10)

and the range is

$$\begin{array}{c} R\left(\tau \right)=\max_{1\leq t\leq \tau }X\left(t,\tau \right)-\min_{1\leq t\leq \tau }X\left(t,\tau \right),\;\tau =1,2,\ldots . \end{array}$$ (11)

The standard deviation is

$$\begin{array}{c} S\left(\tau \right)={\left[\frac{1}{\tau }\sum_{t=1}^{\tau }{\left(X\left(t\right)-<X{>}_{\tau }\right)}^{2}\right]}^{\frac{1}{2}},\;\tau =1,2,\ldots \end{array}$$ (12)

The ratio of range and standard deviation is *R*(*τ*)/*S*(*τ*), and noted as R/S in the following part. After analyzing the statistical rules of R/S, Hurst discovered the following relation:

$$\begin{array}{c} R/S={\left(C\tau \right)}^{H}, \end{array}$$ (13)

where C is a constant, *H* ∈ (0, 1) is called Hurst index, and different *H* means the sequence has the different variation trend. The sequence is random when *H* = 0.5, the later change trend is contrary to the before when *H* ∈ (0, 0.5), and the later trend of the sequence is consistent with the before when *H* > 0.5. So the mutation time can be found according the value of *H*.

### Mann-Kendall method

Mann-Kendall (MK) method is widely used in trend analysis currently. The MK statistic, S, is defined as follows:

$$\begin{array}{c} S=\sum_{i=1}^{n-1}\sum_{j=i+1}^{n}sgn\left(X_{j}-X_{i}\right), \end{array}$$ (14)

where *X*_*i*_, *X*_*j*_ are the sequential data values, *n* is the length of the data set, and

$$\begin{array}{c} sgn\left(\theta \right)=\left\{\begin{array}{cc} 1 & \;\;\;\theta >0,\\ 0 & \;\;\;\theta =0,\\ -1 & \;\;\;\theta <0. \end{array}\right. \end{array}$$

The statistic *S* is approximately normally distributed when *n* ≥ 8, with the mean and the variance as follows:

$$\begin{array}{c} E(S)=0, \end{array}$$ (15)

and

$$\begin{array}{c} V\left(S\right)=\frac{n\left(n-1\right)\left(2n+5\right)-\sum_{i=1}^{n}t_{i}i\left(i-1\right)\left(2i+5\right)}{18}, \end{array}$$ (16)

where *t*_*i*_ is the number of ties of extent *i*.

The standardized test statistic (*Z*) of the MK test and the corresponding P-value (*p*) for the one-tailed test are respectively given by

$$\begin{array}{c} Z=\left\{\begin{array}{cc} \frac{S-1}{\sqrt{Var(S)}} & \;\;S>0,\\ 0 & \;\;S=0,\\ \frac{S+1}{\sqrt{Var(S)}} & \;\;S<0, \end{array}\right. \end{array}$$

and

$$\begin{array}{c} p=0.5-\Phi (|Z|),\;\;(\Phi (|Z|)=\frac{1}{\sqrt{2\pi }}\int_{0}^{\left|Z\right|}e^{-\frac{t^{2}}{2}}dt). \end{array}$$ (17)

If the p-value is small enough, then the trend is quite unlikely to be caused by random sampling. A positive and negative Z value indicates an upward and downward tendency respectively. At the significance level of 0.05, if *p* ≤ 0.05, then the existing trend is considered to be statistically significant.

## Results

### Signal of acceleration of water cycle in Xinjiang

**Fig 2:**
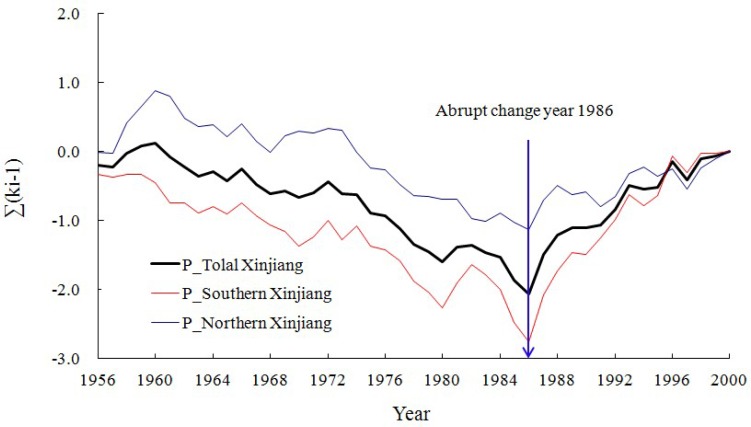
Cumulative curve of modulus coefficient for Xinjiang precipitation from 1956 to 2000. P_−_Total Xinjiang, P_−_Southern Xinjiang and P_−_Northern Xinjiang represent cumulative valuation of modulus coefficient for precipitation in total Xinjiang, southern Xinjiang and northern Xinjiang, respectively.

Precipitation, as the pivotal step of water cycle, is one of the most appropriate index to describe the speed of the water cycle. Fig 2 shows the cumulative valuation of modulus coefficient of Xinjiang precipitation, which indicates that the mutations of precipitation in southern and northern Xinjiang as well the whole Xinjiang appeared in 1986. After 1986, precipitation in Xinjiang increased rapidly from mid-late 1980s to the beginning of the 21st century when global warming was significant. It implied a signal that the local water cycle in Xinjiang was accelerated.

**Fig 3:**
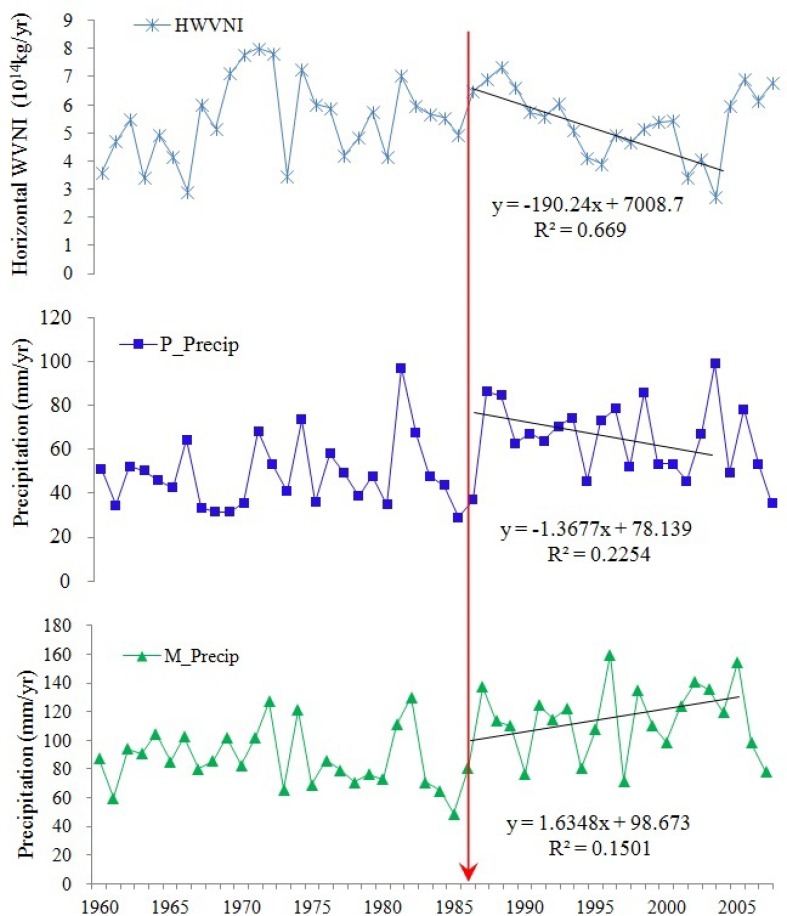
Time series and trend of water cycle elements such as (a) WVNI over Tarim River Basin, (b) precipitation in Tarim plains and (c) precipitation in Tarim mountains from 1960 to 2007.

**Fig 4:**
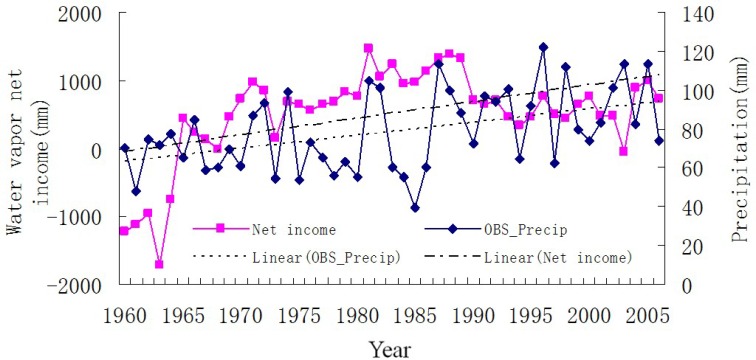
Time series and trend of precipitation and WVNI over Tarim River Basin from 1960 to 2006. After 1986, the WVNI decreased but precipitation in Xinjiang increased rapidly.

Further examination showed that the signal of acceleration did not occur in water cycle all over the Xinjiang. In fact, it mainly occurred over mountains covered by abundant vegetations and glaciers. Taking Tarim River Basin as an example, Fig 3 shows the time series and trend of water cycle elements including WVNI over Tarim River Basin, and precipitation in Tarim Plains and Mountains. The mutations of precipitation in Tarim Mountains and Plains as well as water vapor net income over Tarim region also appeared in 1986. Precipitation in Tarim Plain decreased after the middle of 1980s as well as WVNI over Tarim after a continuous increase from the early 1960s to the middle of 1980s. However, the precipitation in Tarim Mountains increased after the middle of 1980s. The annual average precipitation in mountains (98.59 mm per year), is greater than that in plains (56.10 mm per year). Both precipitation and WVNI over Tarim River Basin exhibit an increasing trend during the period from 1960 to 2006 (Fig 4). However, the cases before and after 1986 are different. More specifically, water vapor revenue over TRB increased at the speed of about 4.79 × 10^12^ kg per year from 1960 to 1986 and decreased at around 1.90 × 10^13^ kg per year from 1986 to 2003, and precipitation in TRB plains decreased at a rate of about 1.37 mm per year, but precipitation in TRB mountains increased at a rate of about 1.63 mm per year after 1986 during the same period (see Fig 3). It indicated that other factors rather than WVNI accelerated the speed of water cycle in Xinjiang.

### Relationship between water cycle and environment factors

**Fig 5:**
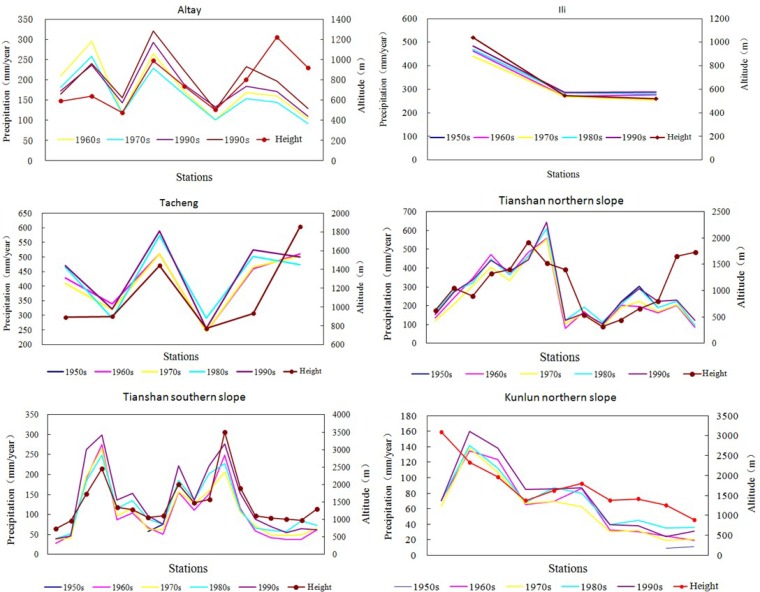
The relationship between precipitation and elevation in Altay, Ili, Tacheng, Tianshan mountains northern slope, Tianshan mountains southern slope and Kunlun mountains northern slope.

**Fig 6:**
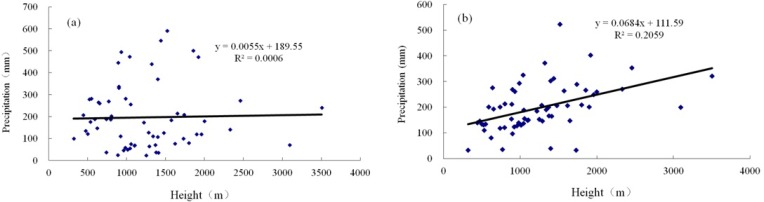
The correlation between precipitation and elevation in total Xinjiang (a) before removal of topography and (b) after removal of topography.

It is shown from Fig 5 that there exists significant correlation between the height and precipitation for stations in the same slope direction, such as Altay, Ili, Tacheng, Tianshan northern slope, Tianshan southern slope, and Kunlun northern slope. The correlation between precipitation and height hardly exists all over Xinjiang (Fig 6a), but they become more related after the removal of topography (Fig 6b). It demonstrated that in the station with higher height, the precipitation is more abundant.

Table 1 shows the transition year of precipitation followed by backward along with some direction associated with the warp or weft. And the difference between precipitation of windward slope and leeward slope is very significant.

**Table 1:** Transition year of station observational precipitation with longitude and latitude in 5 regions of Xinjiang. The stations marked with * and Δ separately represent hydrological stations and meteorological stations.

Region	Station name	Longitude (°E)	Latitude (°N)	Transition Year
Altay	Burqin*	86.86	47.70	1982
	Altay*	88.10	47.82	1983
	Ertai*	90.15	46.05	1985
Ili	Piriqin*	81.48	44.70	1986
	Xinyuan^Δ^	83.30	43.45	1987
Tianshan	Bole^Δ^	82.07	44.90	1984
Northern slope	Qitai^Δ^	89.57	44.02	1985
	Yiwu^Δ^	94.70	43.27	1989
Tianshan southern slope	Hami^Δ^	93.52	42.82	1978
	Shaya^Δ^	82.78	41.23	1980
	Tuoyun^Δ^	75.40	40.52	1985
Kunlun northern slope	Ruoqiang^Δ^	88.17	39.03	1980
	Taxkorgan*	75.23	37.78	1984
	Hetian^Δ^	79.93	37.13	1986

### Physical mechanism behind the acceleration of water cycle

From the above analysis, one can conclude that the speed of water cycle over mountains increases lightly and it is different from plains with global warming. And one question naturally arises: how the physical mechanism of climate changes acts on water cycle. In the following step, we provide a possible physical mechanism behind the acceleration of water cycle.

Water cycle process is one of the energy balance processes of atmospheric system. According to the second law of thermodynamics, atmospheric systems always grow to some balance states through many ways, wherein one way is absorbing (releasing) heat via condensation (evaporation) of water during hydrological process. In a sense, water cycle is the adaptation of atmospheric system to climate change. And the energy transfer direction of water cycle is consistent with the gradient direction of internal air energy as well as temperature. A physical laws (Clausius-Kela Bai Lung relation) determines that water holding capacity of the atmosphere would be increased by about 7% when temperature rise of 1°C, thus global warming causes the increasing of water holding capacity and water vapor content. Consequently, given the same temperature drop range, the mount of reduced atmospheric water vapor content and related precipitation in local with higher temperature would be greater than that with lower temperature. As a result, increasing temperature gradient will naturally speed up the water cycle process. It also can be proved by studies in Ref. [27] that the more local temperature is, the more the proportion of heavy rainfall is.

However, this behavior of atmospheric system will inevitably be controlled by surface water resources. Given a place is rich in water resources on the surface, such as Xinjiang Mountains, the increasing temperature will promote water to evaporate, improve air water vapor content, and accelerate local water cycle.

## Discussion

During the latest several decades, the climate of our planet has been getting warmer and warmer, and makes water cycles to be accelerated in varying degrees for different regions over the world. However, the physical mechanism of global warming increasing the speed of hydrological progress is still uncertain. In this paper, changes in water cycle elements and their relationship with climatic and environmental factors were analyzed. The results show that the increasing precipitation in Xinjiang implied a signal of acceleration of water cycle, and the speed of water cycle is affected by several environmental factors. What’s more, the key factor is temperature. For some areas, such as Xinjiang mountains which with abundant glaciers and vegetation, the risen temperature enlarged the water vapor content and accelerated the speed of water cycle.

We only reveal qualitatively that temperature is one of the key environmental factors. However, the quantitative study on the feedback of water cycle to climate change is not well understood [28], which should be paid more attention since there are more and more heavy rainfall events, even floods occurred in many places all over the world. Besides, our results suggest that global warming speeding up water cycle is depended on the local surface water resources, such as glaciers, rivers, vegetation and so on. Moreover, how they interact and affect the water cycle under the global warming needs further investigation.

In addition, it should be worth pointing out that our results are obtained based on observed and reanalyzed data. However, for a long time precipitation forecast, constructing mathematical models on water cycle are needed which may also provide useful information. As a result, when we need to investigate climate change, it requires to combine mathematical analysis with data processing in the future work.
